# Nonoperative treatment of proximal humerus fractures in the elderly

**DOI:** 10.1007/s00068-025-02912-9

**Published:** 2025-07-07

**Authors:** Sam Razaeian, Christian Krettek

**Affiliations:** 1 Department of Trauma Surgery, Hannover Medical School, Carl-Neuberg-Str. 1, 30625 Hannover, Germany; 2 Department for Trauma, Hand and Reconstructive Surgery, Saarland University, Kirrberger Str. 100, 66421 Homburg, Germany; 3 Department of Trauma Surgery, Hannover Medical School, Hannover Humerus Registry (HHR), TraumaStiftung gGmbH, Carl-Neuberg-Str. 1, 30625 Hannover, Germany

**Keywords:** Proximal humerus fracture, Nonoperative treatment, Conservative, Evidence-based, Elderly, Treatment algorithm

## Abstract

Proximal humerus fractures are common and occur in approximately 70% of the cases beyond the age of 60 with a peak incidence over the age of 80. A number of randomized studies and meta-analyses have so far failed to demonstrate the superiority of surgery over nonoperative treatment in the elderly. In addition, according to the latest systematic Cochrane Review, evidence still reveals that surgery might be associated with higher complication, and revision surgery rates. However, it remains unclear how exactly this evidence out of “controlled” trial conditions can be incorporated into a decision aid suitable for everyday clinical practice. The following review article aims to present a possible treatment algorithm that takes current best evidence into account, and provides guidance on how this steadily increasing type of injury might be successfully treated nonoperatively in the elderly in daily clinical practice.

## Introduction

*“[…] it would be imprudent to restore to the joint its natural form, even were it in our power to accomplish it, for we would thus materially diminish the chance of the occurrence of osseous consolidation … but the prudent surgeon will never omit to announce to the patient that a certain degree of impairment of the motions of the joint will be a permanent result of the injury.”* (Robert William Smith 1847) [1, 2].

Robert William Smith (1807–1873), Irish surgical pioneer, anatomist, and first professor for surgery at the renowned Trinity College, founded in 1847, made a name for himself with the Smith fracture of the distal radius [3], but also investigated on another equally common fracture in the elderly, whose management is still subject of controversy even 150 years later [4].

Proximal humerus fractures (PHFs) are common and occur in approximately 70% of the cases beyond the age of 60 with a peak incidence over the age of 80 [5].

It is assumed that the majority of these fractures can be treated nonoperatively with good results [5–7]. This estimation might be independent of epidemiologic inconsistencies in the literature regarding the actual amount of displaced PHFs, but results from applying current best evidence on the proportion of actually affected age groups [7–9]. That evidence of the last two decades has not been able to demonstrate functional superiority of surgery over nonoperative treatment [10–17]. In addition, in nonoperatively treated patients, evidence suggests lower risks of complications and revision surgery in the elderly [12]. Nevertheless, there are large differences in treatment, with surgical rates over 40% in some nations, and institutions, highlighting the inconsistent management and lack of consensus in treatment of this common injury [4, 6, 7, 18, 19].

In Germany, for example, only 55.6% of patients over 65 years of age or older were treated nonoperatively in 2021 according to a recent study analyzing outpatient and inpatient billing data from the second largest health insurance company [20].

In that country, there are already around 24.4 million people over the age of 60, making up one third of total population, with a predicted increase by around 3 million in this age group until 2040 [21]. Nevertheless, there is no current valid national guideline from professional societies on PHFs for over 2 years in that country, and this is likely not expected to change until the end of 2025 [4]. Considering these epidemiological developments, which do not only apply to that country [6, 7], management of this injury will become increasingly important in the future also in socio-economic terms.

The following review article aims to present a possible treatment algorithm that takes current best evidence into account, and provides guidance on how this steadily increasing type of injury might be successfully treated nonoperatively in the elderly in daily clinical practice outside of controlled study conditions [4].

## Basic principles of nonoperative treatment of PHF

**Fig. 1:**
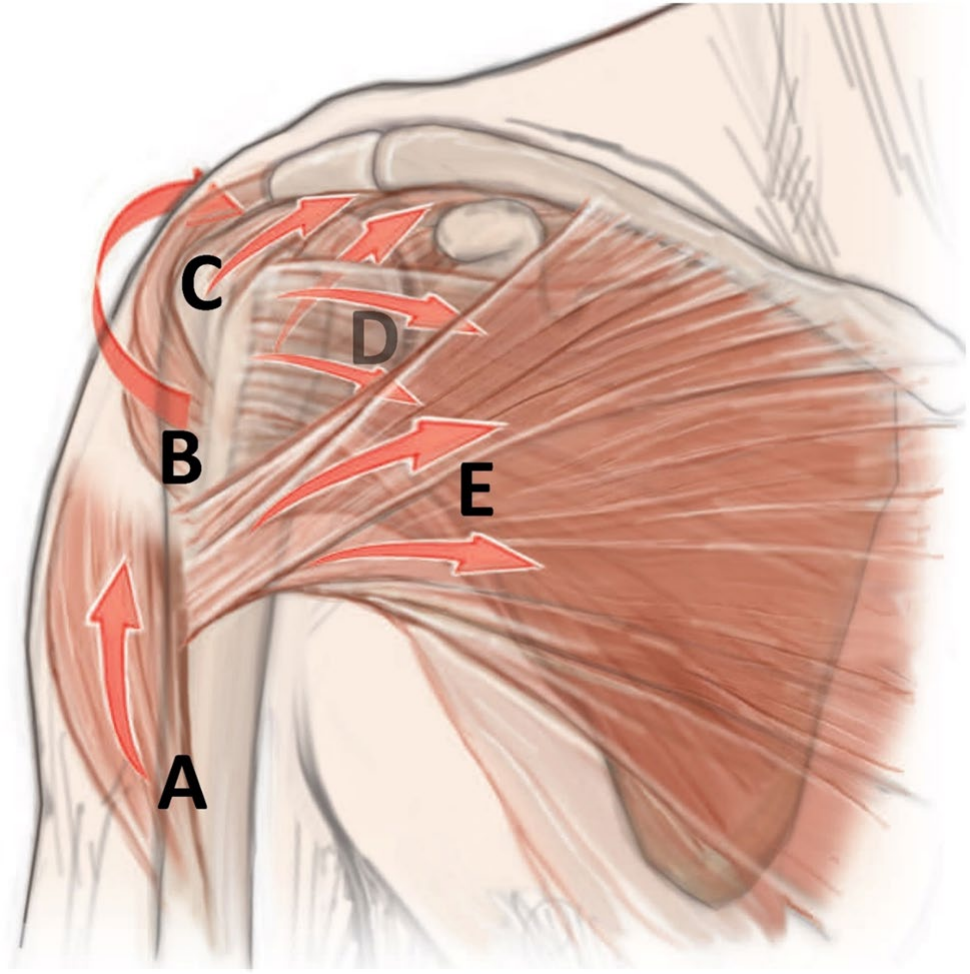
Schematic illustration of “deforming” muscle forces acting on a proximal humerus fracture. M. deltoideus (**A**), M. infraspintatus + teres minor (**B**), M. supraspinatus (**C**), M. subscapularis (**D**), M. pectoralis major (**E**). Reprinted from *“Algorithmus zur konservativen Behandlung von proximalen Humerusfrakturen”* (https://link.springer.com/article/10.1007/s11678-022-00702-y, Copyright© 2022, Razaeian) under the terms of the Creative Commons CC BY 4.0 license (http://creativecommons.org/licenses/by/4.0/deed.de)

The syllable “non” in the word “nonoperative” falsely suggests a neglect or even a simple omission of a necessary treatment [22]. However, this treatment can turn out to be much more complex than surgery. Nonoperative treatment is based on the understanding of the so-called “deforming forces” acting on the fracture (Fig. 1), the influence of gravity, and the targeted use of medical aids as bandages and orthosis, taking fracture morphology into account [4].

In the 1860 s, Lucas-Championnière elaborated a functional treatment principle based on the observation, that healing of PHF in a correct anatomical position is not necessary to obtain good function [4, 23].

Several later authors confirmed this hypothesis, which breaks with a fundamental principle of reconstructive trauma surgery. Same as DePalma und Cautilli [24], Mills and Horne [25], also Young and Wallace stated that radiographic appearance did not correlate with clinical outcome in the elderly [1, 4, 26, 27]. With regard to fracture reduction accuracy would be not essential due to the multiaxial nature of the glenohumeral joint as well as presence of scapulothoracic movement permitting considerable deformity [23, 26]. Especially in elderly patients some authors suggested, that almost any degree of angulation could be tolerated [4, 28].

**Fig. 2:**
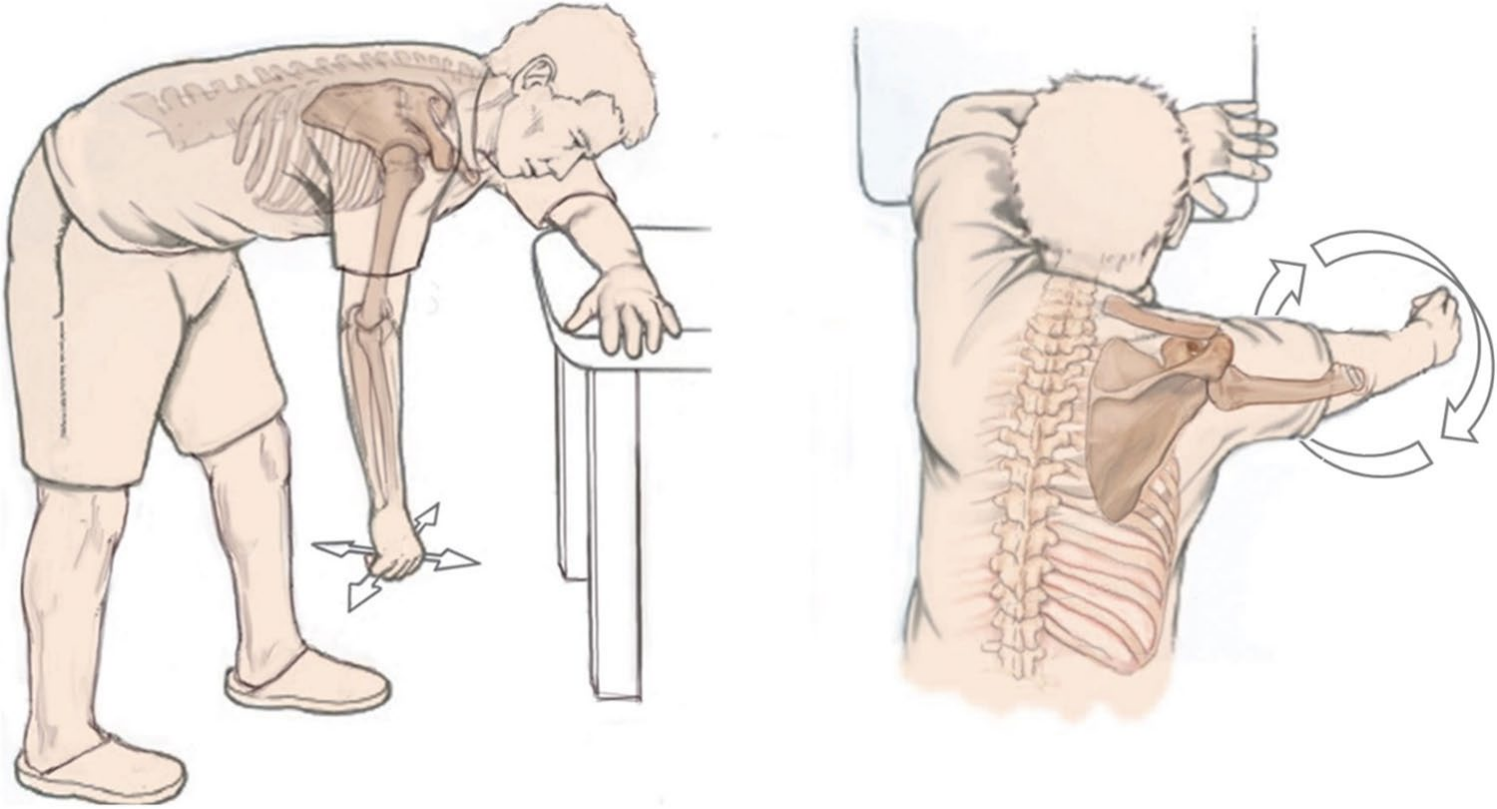
Illustration of the pendulum exercises according to Codman in the so-called “hanging-loose position” [29]. The left image shows anteversion/retroversion and abduction/adduction movements, while the right image demonstrates circling movements. A small weight or a lead wrist cuff can increase gravity effect. Reprinted with changes from *“Algorithmus zur konservativen Behandlung von proximalen Humerusfrakturen”* (https://link.springer.com/article/10.1007/s11678-022-00702-y, Copyright© 2022, Razaeian) under the terms of the Creative Commons CC BY 4.0 license (http://creativecommons.org/licenses/by/4.0/deed.de)

Some authors have also pointed out another phenomenon that has been less frequently discussed to date, that for example a displaced 4-part fracture according to Neer spontaneously appears as a slightly minimally displaced 1-part fracture only one week after injury [29, 30]. One of the oldest hypotheses states that gravity is a driving force for this self-reducing potential [4, 23, 29]. DePalma and Cautilli [24] and others hypothesized decades ago that the weight of the affected arm of about 4–7 kg might be sufficient in many cases to correct most fractures [4, 23]. Codman's pendulum exercises in the so-called “hanging-loose position” are based on this theory as a fundamental part of follow-up treatment (Fig. 2) [29], underlining the importance of early mobilization, and clarifying bedriddenness, for example due to concomitant injuries or sarcopenia in otherwise cognitively unimpaired patients, as a challenge for nonoperative treatment [4, 23].

## Indication

*“There is one very striking thing about fractures of the humerus, and that is that most cases eventually recover pretty good use of their shoulders in spite of any kind of treatment.”* Ernest Amory Codman (1934) [31, 32].

A number of randomized studies and meta-analyses have so far failed to demonstrate the superiority of surgery over nonoperative treatment in the elderly (Table 1) [10–17]. In addition, according to the latest systematic Cochrane Review, evidence still reveals that surgery might be associated with higher complication, and revision surgery rates [9, 12]. However, it remains unclear how exactly this evidence out of “controlled” trial conditions can be incorporated into a decision aid suitable for everyday clinical practice [4]. Unfortunately, the large imbalance in the literature between a fraction of less than 5% of papers on nonoperative treatment on the one hand and more than 60% of papers on surgical treatments on the other, does not seem to contribute to solve this problem [40–42]. The authors believe that the same is true for congresses, educational meetings, and textbooks.

**Table 1:** Overview of randomized controlled trials comparing nonoperative (NO) versus operative (OT) treatment in a predominantly elderly patient population

Author, journal, and year	Mean age in years	Number of patients	Number of fractures according to Neer classification	Surgical method	Final follow up in months	Outcome scores	Statistical outcome difference^1^	Secondary surgery rate after NO	Secondary surgery rate after OT
Olerud, JSES 2011 [33]	77	55	4-part: 55	HA	24	CS, DASH, ROM, EQ-5D, VAS	Significant difference favoring HA regarding EQ-5D (0.81 vs. 0.65, *p* = 0.02). Nonsignificant trend for better DASH (*p* = 0.25) and VAS (*p* = 0.17) in HA group. No significant difference regarding CS and ROM	4%	11%
Olerud, JSES 2011 [34]	74	60	3-part: 60	ORIF	24	CS, DASH, ROM, EQ-5D, VAS score (0–100 points)	Nonsignificant trend favoring ORIF regarding CS (61 vs. 58 points, *p* = 0.64), DASH (26 vs. 35, *p* = 0.19), EQ-5D (0.70 vs. 0.59, *p* = 0.26), and ROM (mean flexion: 120° vs. 111°, *p* = 0.36; mean abduction: 114° vs. 106°, *p* = 0.28). No significant difference in VAS pain score (20 vs. 17 points, *p* = 0.94)	3%	30%
Fjalestad, JOT 2012 [35]	73	48	3-part^2^: 26^3^ 4-part^2^: 24^3^	ORIF	12	modified ASES self evaluation, CS	No significant difference in ASES. Nonsignificant trend for better CS by 2.4 points in NO group (*p* = 0.62)	4%	17.4%
Fjalestad, EJOST 2014 [36]	73	42	3-part^2^: 26^3^ 4-part^2^: 24^3^	ORIF	24	modified ASES self evaluation, 15D	No significant difference	4%	4.4%
Boons, CORR 2012 [37]	76 to 80^4^	50	4-part: 50	HA	12	CS, SST, VAS pain, VAS disability, abduction strength	No significant difference except for better abduction strength in NO group (*p* = 0.008)	4%	4%
Rangan, JAMA 2015 [38]	66	250	1-part: 18 2-part: 128 3-part: 93 4-part: 11	ORIF	24	OSS, SF-12	No significant difference	9%	9%
Launonen, PLOS Med 2019 [39]	72 to 73^4^	88	2-part: 88	ORIF	24	CS, DASH, OSS, EQ-5D(−3L), 15D, VAS	No significant difference	0%	6.8%
Lopiz, JSES 2019 [14]	84	59	3-part: 9 4-part: 50	RSA	12	CS, DASH, SF-12, EQ-5D, EQ-VAS, VAS	No significant difference except for better VAS in RSA group (0.9 vs. 1.6, *p* = 0.011)	0%	0%
Launonen, PLOS Med 2023 [15]	69 to 70^4^	160	3-part: 99 4-part: 61	ORIF and HA	24	CS, DASH, OSS, EQ-5D(−3L), 15D, VAS	No significant difference	3.8%	16.3%
Miquel, JSES 2024 [22]	77	66	3-part: 45^3^ 4-part: 36^3^	RSA	12	CS, adj. CS	Significant difference favoring surgery, but without reaching MCID	0%	3.2%

*HA* Hemiarthroplasty, *ORIF* Open reduction and internal plate fixation (mainly locking plate), *RSA* Reverse shoulder arthroplasty. *Adj. CS* adjusted Constant score, *ASES* American Shoulder and Elbow Surgeons-self evaluation score, *CS* Constant score, *DASH *Disabilities of the Arm, Shoulder and Hand score, *OSS* Oxford shoulder score, *ROM* range of motion, *SF-12* Short-Form-Health Survey, *SST* Simple shoulder test, *VAS* Visual analogue scale, *15-D* 15-dimensional standardized, self-administered measure of health-related quality of life. *MCID* Minimal clinically important difference

^1^Level of significance *p* < 0.05. ^2^Classified according to AO/OTA 2007 system. ^3^Fracture type distribution only for initially included cohort before lost-to-follow-ups reported. ^4^No mean age for total cohort reported

**Fig. 3:**
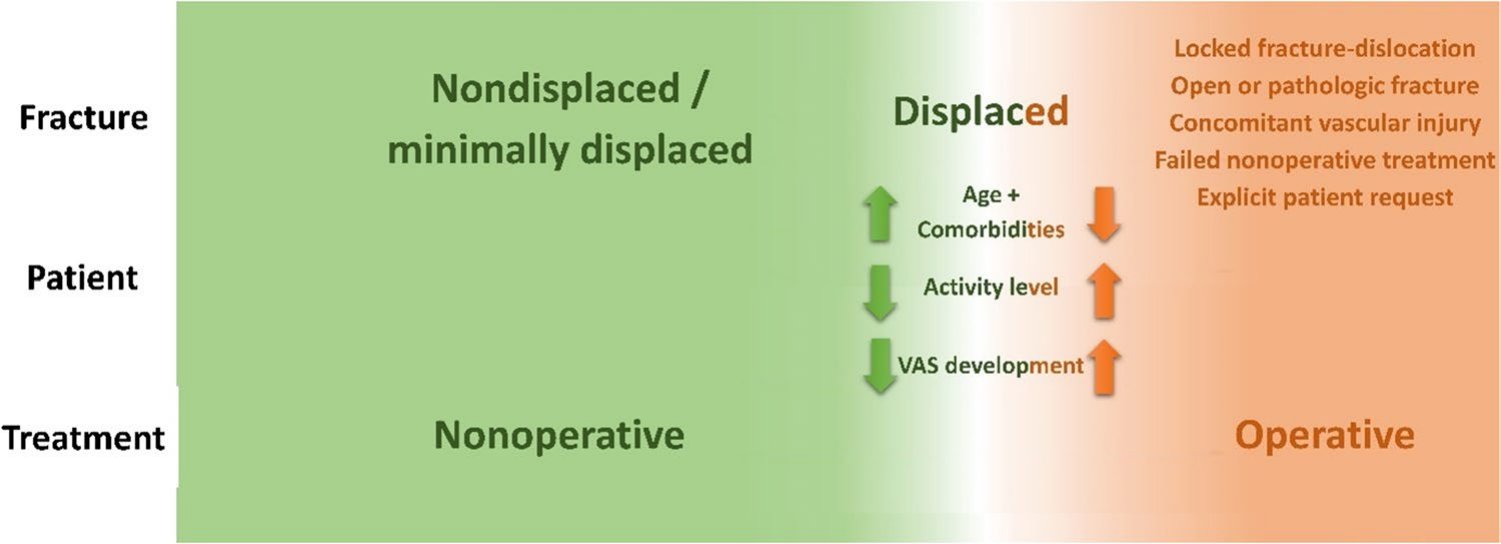
Schematic treatment algorithm according to Krettek et al. [4, 43] for treatment of proximal humerus fractures in the elderly. *VAS* visual analog scale. Reprinted with changes from *“Algorithmus zur konservativen Behandlung von proximalen Humerusfrakturen”* (https://link.springer.com/article/10.1007/s11678-022-00702-y, Copyright© 2022, Razaeian) under the terms of the Creative Commons CC BY 4.0 license (http://creativecommons.org/licenses/by/4.0/deed.de)

Figure 3 shows a scheme of a possible decision aid that has proven itself as an algorithm at the authors’ institute between 01/2016 and 09/2021. Usual fix cut-off values for surgical treatment such as age (< 65 vs. ≥ 65 years), fracture morphology, bone quality or degree of dislocation do not exist in this algorithm [4]. Instead, apart from a few absolute indications for surgery, every fracture is primarily treated nonoperatively. For example, head-split fractures are listed in the scheme as an absolute contraindication for nonoperative treatment as this rare fracture type is not covered by the evidence of the current Cochrane Review due to nonexistent RCTs. However, according to the authors’ observation, nonoperative treatment may also be an option for this fracture type in selected cases in the elderly, particularly if one considers the limited outcomes after surgical treatment. In a retrospective study, 30 patients with head-split fractures were examined with a mean age of 63 years and mean follow-up of 49 months [44]. The majority were treated with plate osteosynthesis (ORIF, *n* = 24), 4 patients with reverse shoulder arthroplasty (RSA), and 2 with hemiarthroplasty (HA). The overall complication rate was 83% (ORIF: 88%, RSA: 75%, and HA 50%), and revision surgery rate 29% after ORIF, while no revision was performed after RSA, and HA [44]. Nevertheless, clinical outcomes were limited among all three types of surgery with a mean Subjective shoulder value of 58%, and mean adjusted Constant score of 46 points [44].

In the case of displaced PHFs, patient-related factors (age, comorbidity, degree of activity or functional demand, patient's request, and development of pain level) are included in decision-making process of the scheme [4].

Considering that the combination of a displaced PHF in a young, highly active patient in a larger trauma center with a corresponding patient flow is a rather rare scenario, it is plausible that the majority of PHFs might be treated nonoperatively with the aid of this scheme [4].

## Diagnostic imaging

Conventional X-ray in at least two planes (AP- and Y-view) is considered as a necessary basic diagnostic examination, and CT-scan as optional also according to the ESTES recommendations [45].

Possible indications for the latter include a better assessment of the tuberosities, articular surface (e.g., suspicion of head-split morphology), glenohumeral centering, assessment of bone density, and the advantage of three-dimensional (3D) reconstruction [4, 45].

The actual value of additional cost- and time-intensive CT-scan in the elderly is debatable [4].

At the authors’ institute, both imaging modalities (X-ray and CT) were routinely used to classify the fracture morphology purely descriptively (varus/valgus, impacted/distracted, metaphyseal integrity, shaft translation), and in order to select the most suitable reduction as well as retention aids [4]. This approach must consider that CT imaging is performed on a supine patient [4, 46].

From the authors’ point of view, it is recommended that X-rays, and any fluoroscopic imaging are performed in an upright position (sitting, preferably standing), if possible [4, 46].

**Fig. 4:**
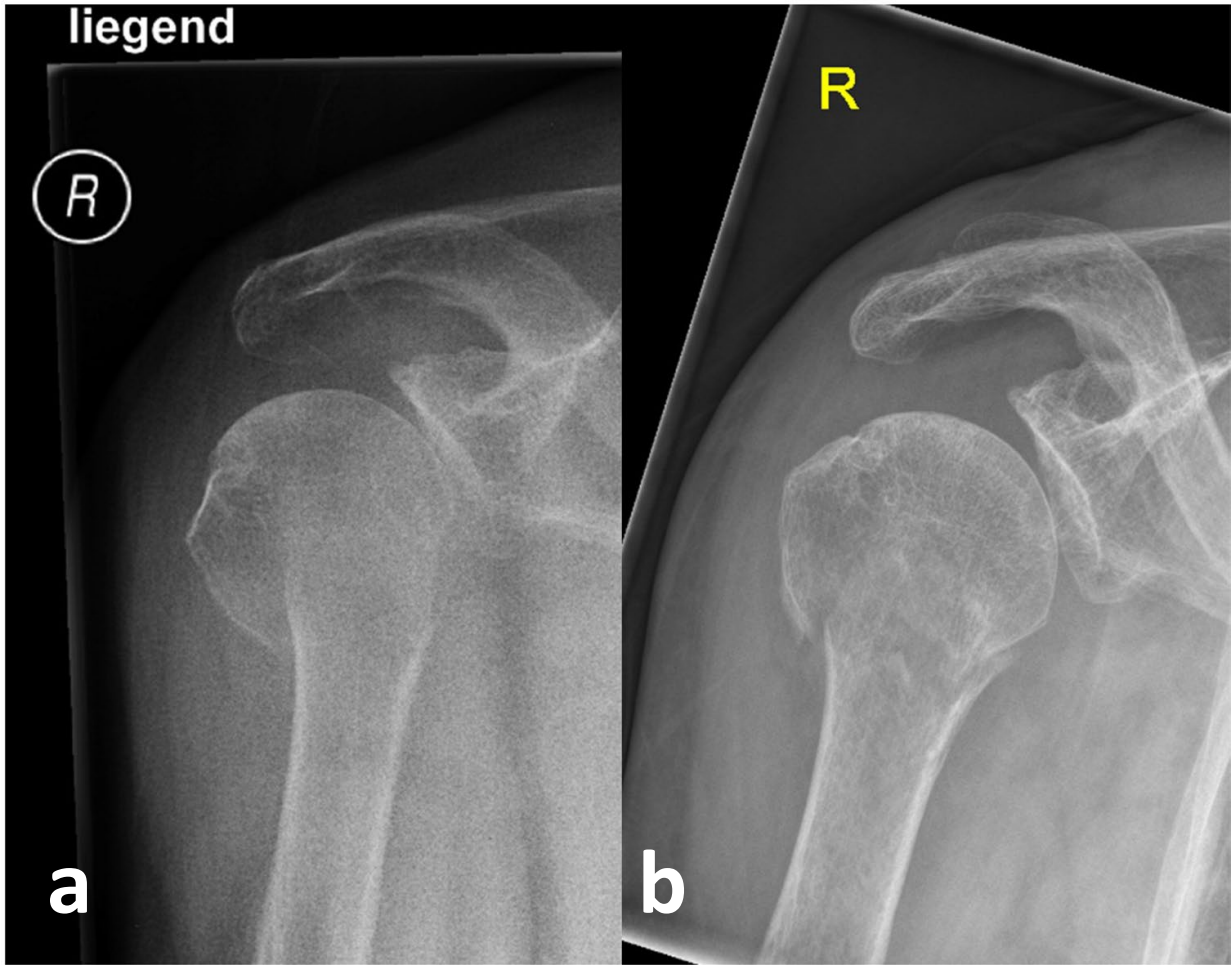
AP radiograph of an 85-year-old female patient on the day of injury in a lying supine position (**a**) and about 3 weeks later in a standing position with almost complete self-reducing of the presumed displacement that existed on the day of injury (**b**). Reprinted from *“Algorithmus zur konservativen Behandlung von proximalen Humerusfrakturen”* (https://link.springer.com/article/10.1007/s11678-022-00702-y, Copyright© 2022, Razaeian) under the terms of the Creative Commons CC BY 4.0 license (http://creativecommons.org/licenses/by/4.0/deed.de)

This might be essential not only to assess the possible self-reducing potential of the fracture, but also to avoid overestimating the initial assumed degree of displacement (Fig. 4) [4, 46].

**Fig. 5:**
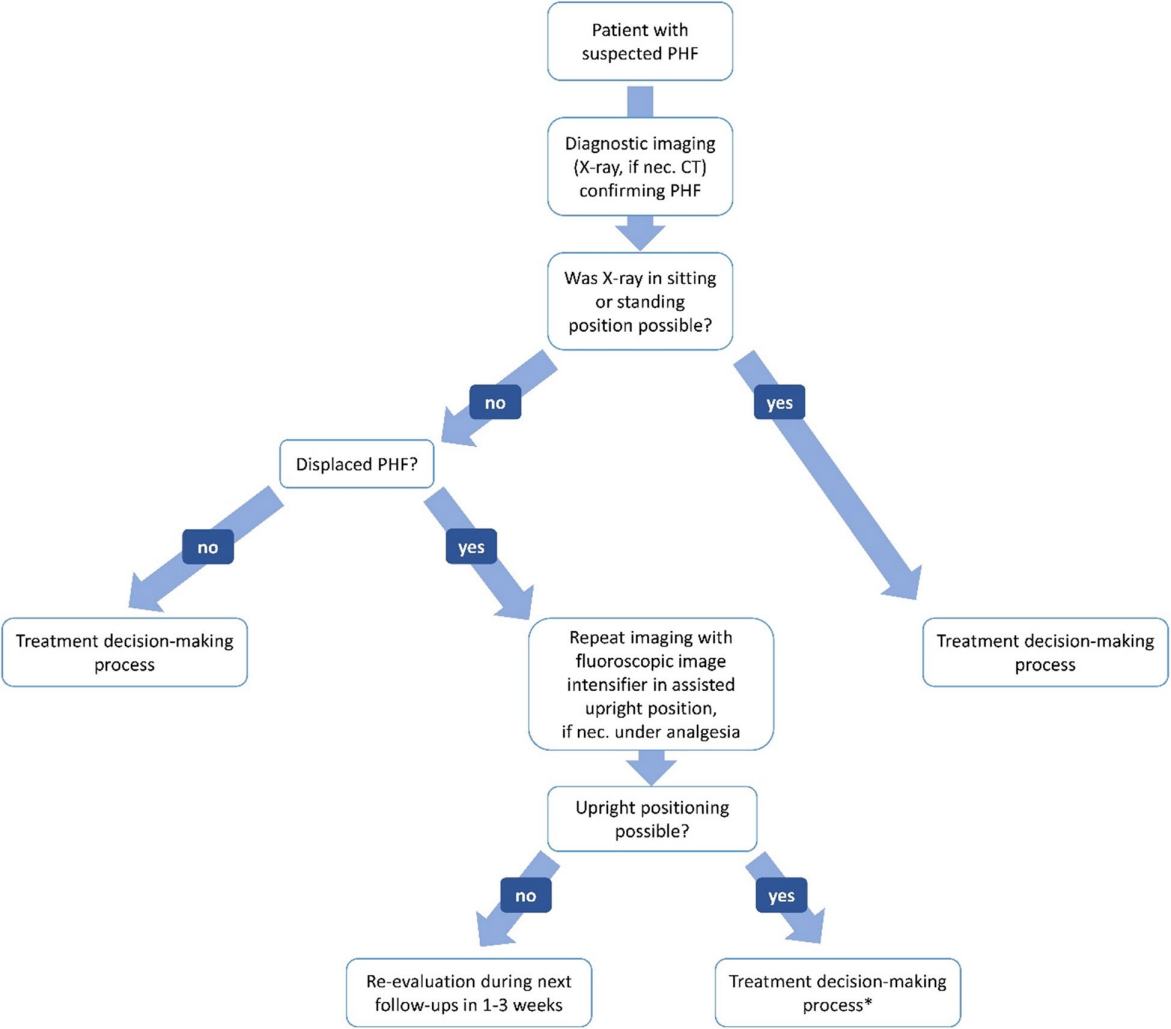
Recommended procedure before treatment decision. *Further self-reducing potential should be taken into account. Reprinted from *“Self-Reduction in Proximal Humerus Fractures through Upright Patient Positioning: Is It up to Gravity?”* (https://doi.org/10.3390/diagnostics12092096, Copyright© 2022, Razaeian, Licensee MDPI, Basel, Switzerland) under the terms of the Creative Commons CC BY 4.0 license (http://creativecommons.org/licenses/by/4.0/deed.de)

If this is not feasible on the day of the accident due to high pain level, it should be repeated during the further course before any treatment decision (Fig. 5) [4, 46].

## Closed reduction, medical aids and aftercare for nonoperative treatment

*“Via sanationis, partim ad naturam attinet, quæ callo fracturam agglutinat; partim ad medicum qui impedimenta submovet, & ossa componit.”* Vidus Vidius (1596) [32, 47]

*“The path to healing is partly achieved by nature, which unites the fracture with callus, and partly by the physician, who removes obstacles and sets the bones.”* (Own translation)

The most suitable medical aids (bandages, orthosis) for closed fracture reduction and retention are selected considering fracture morphology, and deforming muscle forces acting on the fracture [4]. In addition, one should bear in mind that closed manipulation attempts are limited only to the distal segment of the fractured proximal humerus (humeral shaft) (Fig. 1) [4]. The position of small main fragments (greater and lesser tuberosity, humeral head) is difficult or impossible to manipulate by closed means. In case of severe displacement, it can be useful to perform closed reduction and apply those aids under fluoroscopic control on patients in an upright sitting, preferably standing position [4, 46].

**Fig. 6:**
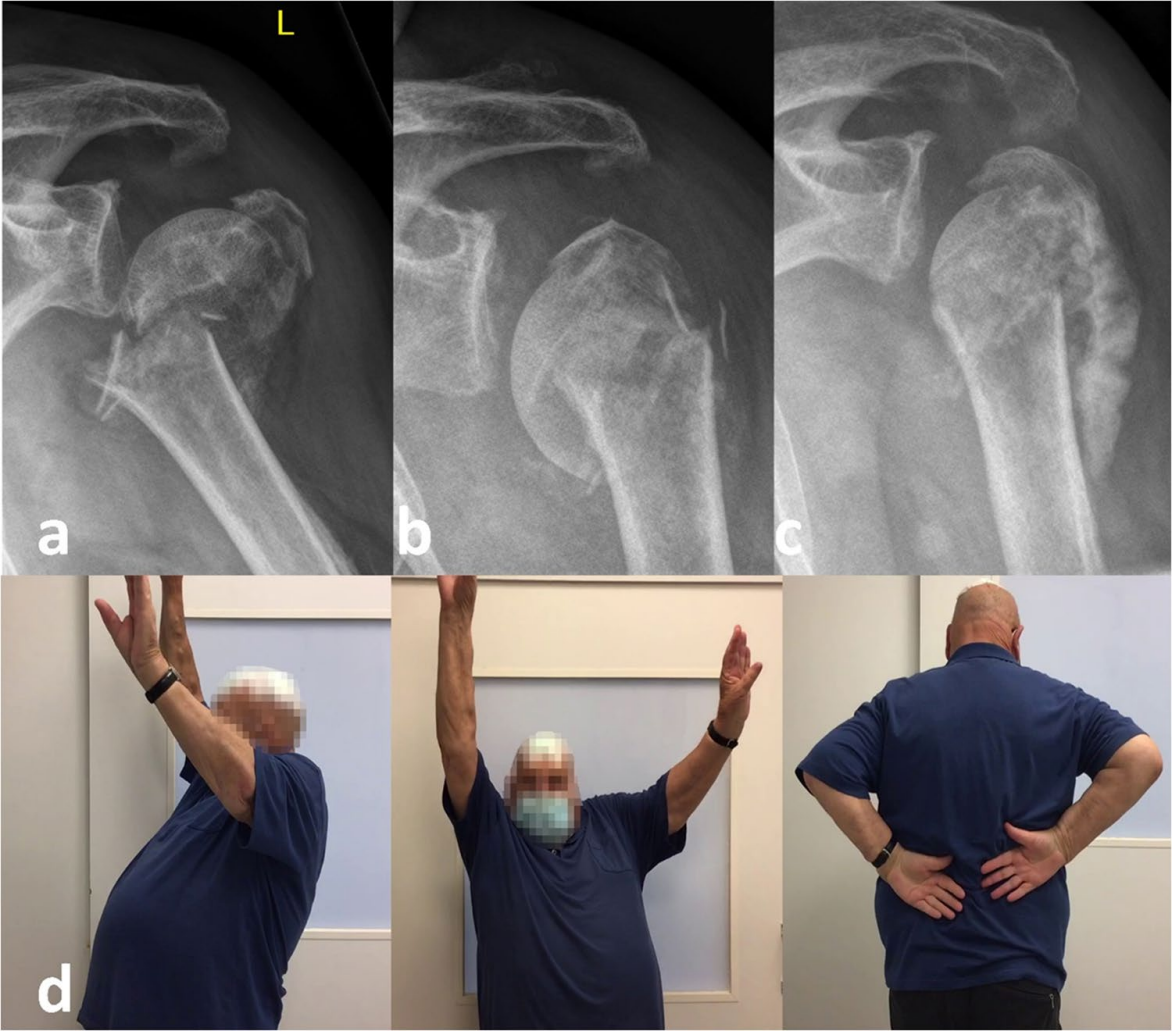
AP radiographs of a 79-year-old patient with considerable medial displacement of humeral shaft with metaphyseal comminution and significant valgus displacement of the humeral head (**a**). Without any reduction maneuvers or medical aids, orthograde self-reducing of the humeral shaft was observed after 6 weeks, however also secondary varus displacement of the humeral head (**b**). Despite this, nonperative treatment was continued with decreasing pain level. After 6 months fracture healing in nonanatomic position (**c**) with nevertheless good functional outcome (subjective shoulder score: 80%, absolute Constant Score: 75 out of 100 points, no pain; **d**, **e**, **f**). Reprinted with changes from *“Algorithmus zur konservativen Behandlung von proximalen Humerusfrakturen”* (https://link.springer.com/article/10.1007/s11678-022-00702-y, Copyright© 2022, Razaeian) under the terms of the Creative Commons CC BY 4.0 license (http://creativecommons.org/licenses/by/4.0/deed.de)

Experience has proven that optimal reduction and retention using the following aids as well as healing in an anatomic position is not that essential in the elderly as in young, active patients who cannot compensate residual bony deformities (Fig. 6) [4].

The following tools have proven to be helpful.

### Arm sling/gilchrist bandage

**Fig. 7:**
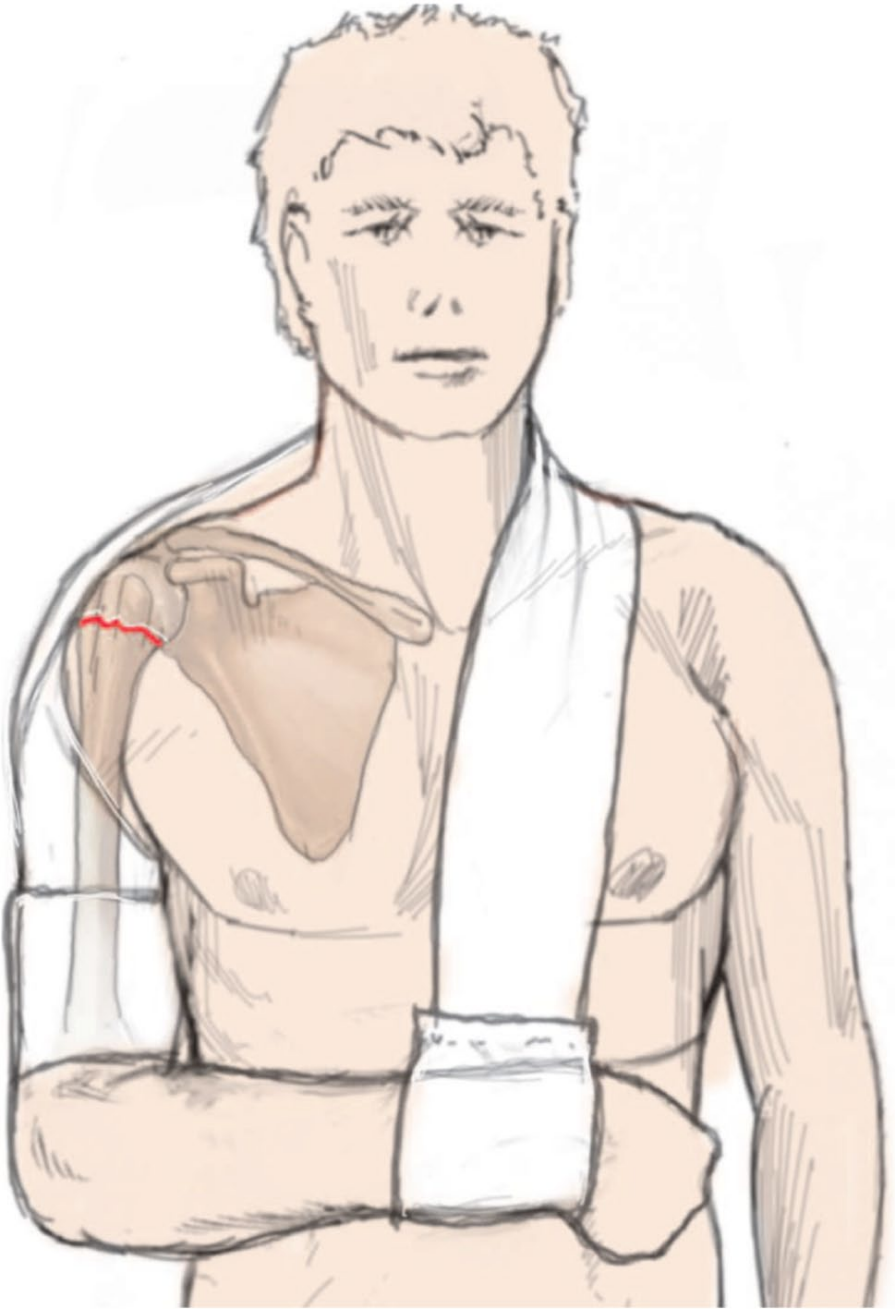
Patient with an arm sling. In this commercially available version, the upper arm and forearm are free and not encircled by the bandage as would be the case with the classic Gilchrist bandage. Reprinted from *“Algorithmus zur konservativen Behandlung von proximalen Humerusfrakturen”* (https://link.springer.com/article/10.1007/s11678-022-00702-y, Copyright© 2022, Razaeian) under the terms of the Creative Commons CC BY 4.0 license (http://creativecommons.org/licenses/by/4.0/deed.de)

**Fig. 8:**
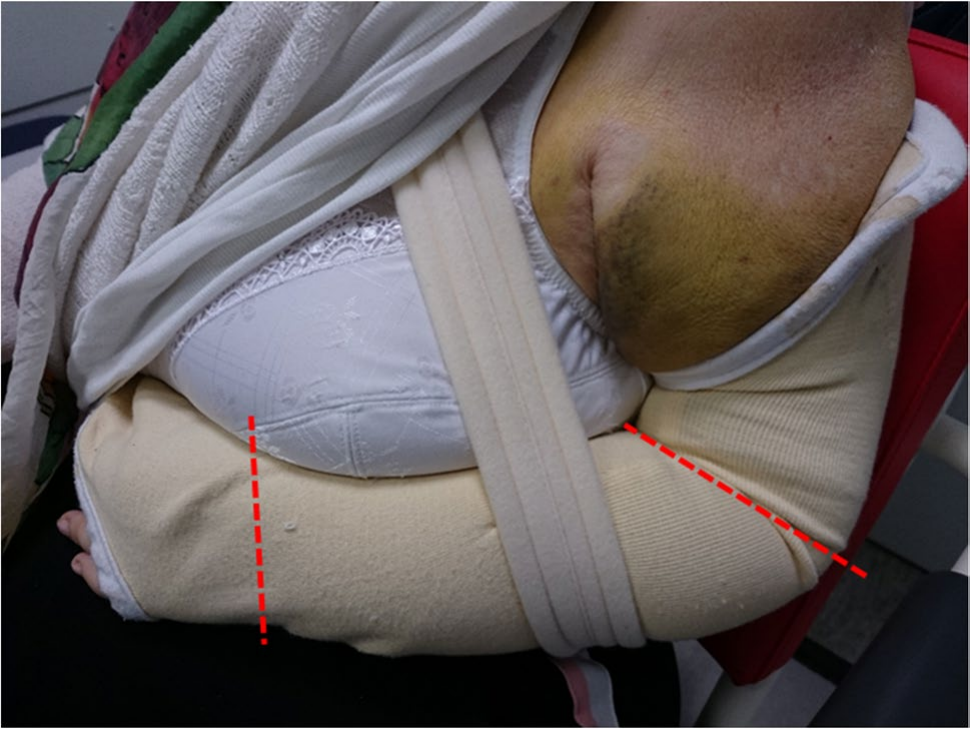
Negative example of a Gilchrist bandage that is too small. Wrist and fingers should always be free (cut). This commercially available model often needs to be individualized and adapted to the patient's need. An incision of the bandage at the level of the dashed red lines can prevent a painful cuttting into the at the level of the elbow groove and offering considerably more comfort through free use of the hand/fingers. Reprinted from *“Algorithmus zur konservativen Behandlung von proximalen Humerusfrakturen”* (https://link.springer.com/article/10.1007/s11678-022-00702-y, Copyright© 2022, Razaeian) under the terms of the Creative Commons CC BY 4.0 license (http://creativecommons.org/licenses/by/4.0/deed.de)

The arm sling is useful for simple fractures and serves primarily to reduce pain (Fig. 7). It can also be a basis for a variety of modifications in order to hold a reduced fracture. It should be taken care to select the correct size when applying it. In addition, in some sling versions as the Gilchrist bandage, it has proven useful to incise the bandage at the level of the elbow groove and at wrist level in order to avoid compromising usually swollen soft tissues due to hematoma/swelling and to give the patient more freedom of movement (Fig. 8) [4].

### Plaster-reinforced gilchrist bandage

**Fig. 9:**
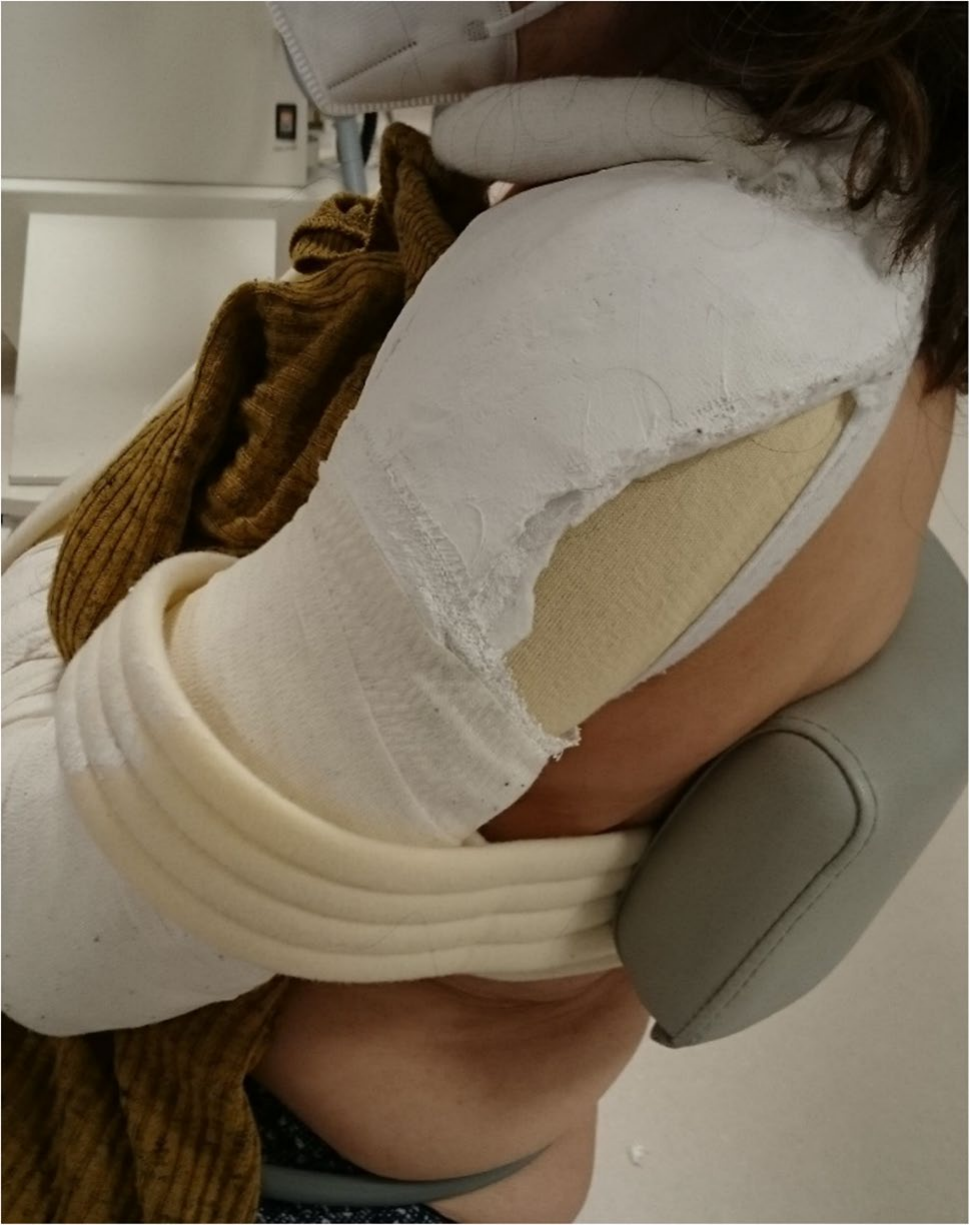
Patient with plaster-reinforced Gilchrist bandage. Reprinted from *“Algorithmus zur konservativen Behandlung von proximalen Humerusfrakturen”* (https://link.springer.com/article/10.1007/s11678-022-00702-y, Copyright© 2022, Razaeian) under the terms of the Creative Commons CC BY 4.0 license (http://creativecommons.org/licenses/by/4.0/deed.de)

If the fracture is unstable (e.g., metaphyseal comminuted zone or combined PHF with humeral shaft fracture), additional plaster reinforcement might be helpful and provide more stability, and thus pain relief (Fig. 9). Care should be taken to avoid pressure ulcers due to the cast in the elderly [4].

### Abduction orthosis

**Fig. 10:**
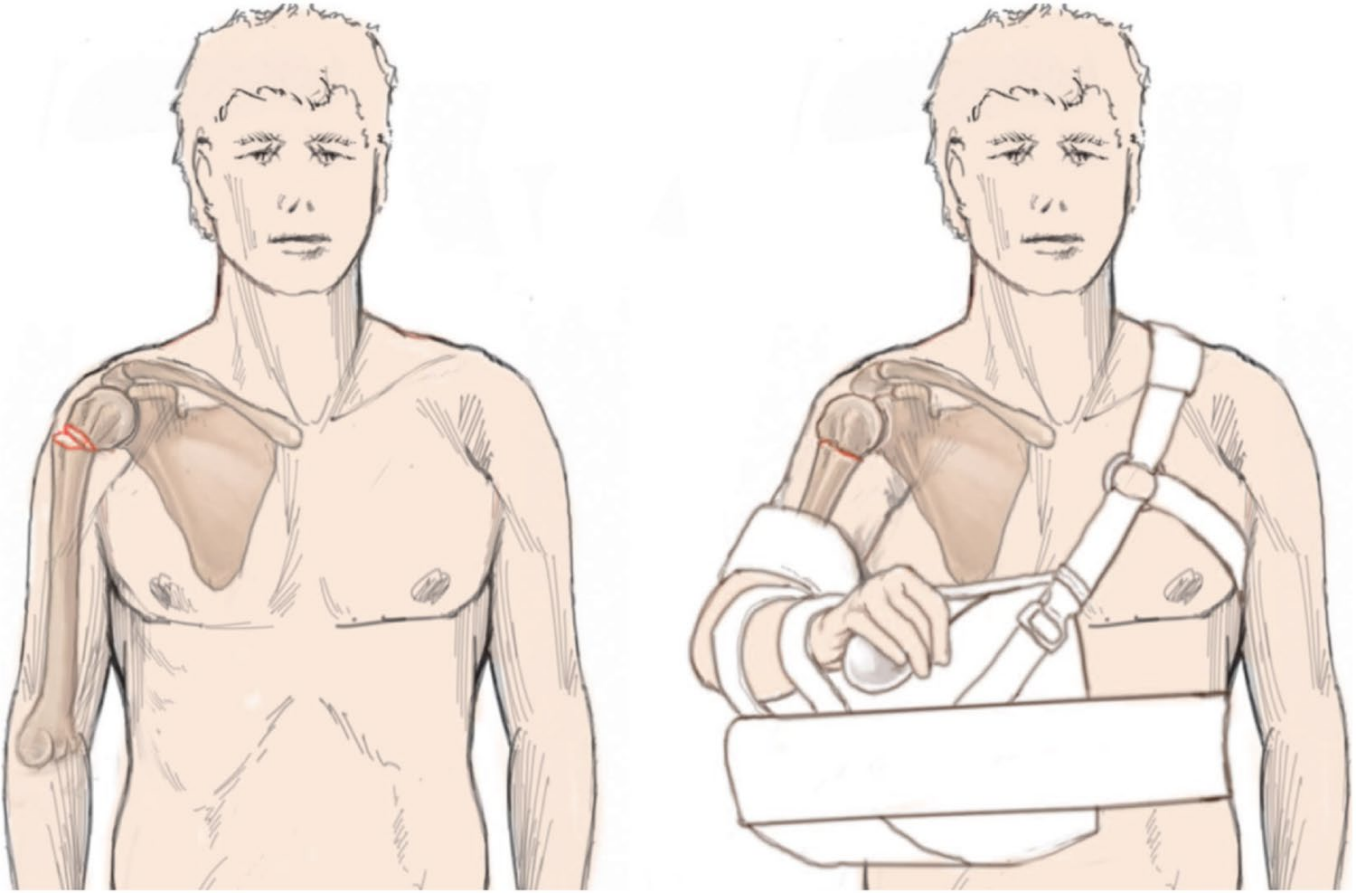
Patient with varus displaced fracture before (left) and after (right) application of the abduction orthosis. In this example, the humeral shaft is adjusted to the displaced humeral head fragment by abduction of the arm. Reprinted from *“Algorithmus zur konservativen Behandlung von proximalen Humerusfrakturen”* (https://link.springer.com/article/10.1007/s11678-022-00702-y, Copyright© 2022, Razaeian) under the terms of the Creative Commons CC BY 4.0 license (http://creativecommons.org/licenses/by/4.0/deed.de)

In the case of varus-displaced fractures and/or greater tuberosity involvement, an abduction orthosis might be used. As only the position of the humeral shaft can be manipulated, the shaft is approximated to the displaced humeral head and/or greater tuberosity fragment by applying the orthosis (Fig. 10) [4].

### Axillary roll

**Fig. 11:**
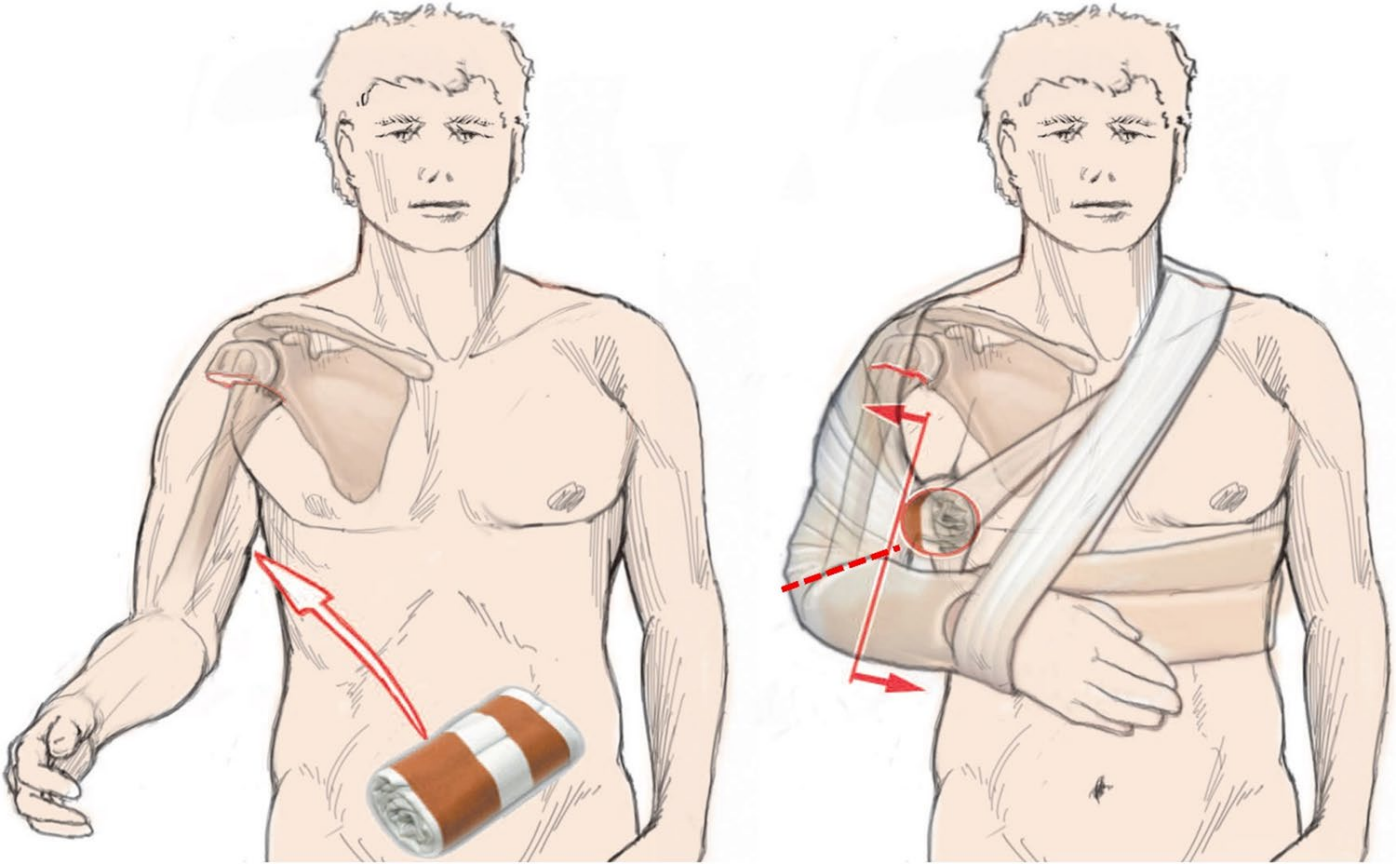
Patient with medial shaft translation before (left) and after (right) application of a Gilchrist bandage and axillary roll according to Krettek et al. [4, 43, 48]. This acts as a hypomochlion to counteract the medial shaft translation by the pull of the pectoralis major muscle. An incision of the bandage at the level of the dashed red line can prevent a painful cuttting into the elbow groove and offering considerably more comfort. Reprinted with changes from *“Algorithmus zur konservativen Behandlung von proximalen Humerusfrakturen”* (https://link.springer.com/article/10.1007/s11678-022-00702-y, Copyright© 2022, Razaeian) under the terms of the Creative Commons CC BY 4.0 license (http://creativecommons.org/licenses/by/4.0/deed.de)

**Fig. 12:**
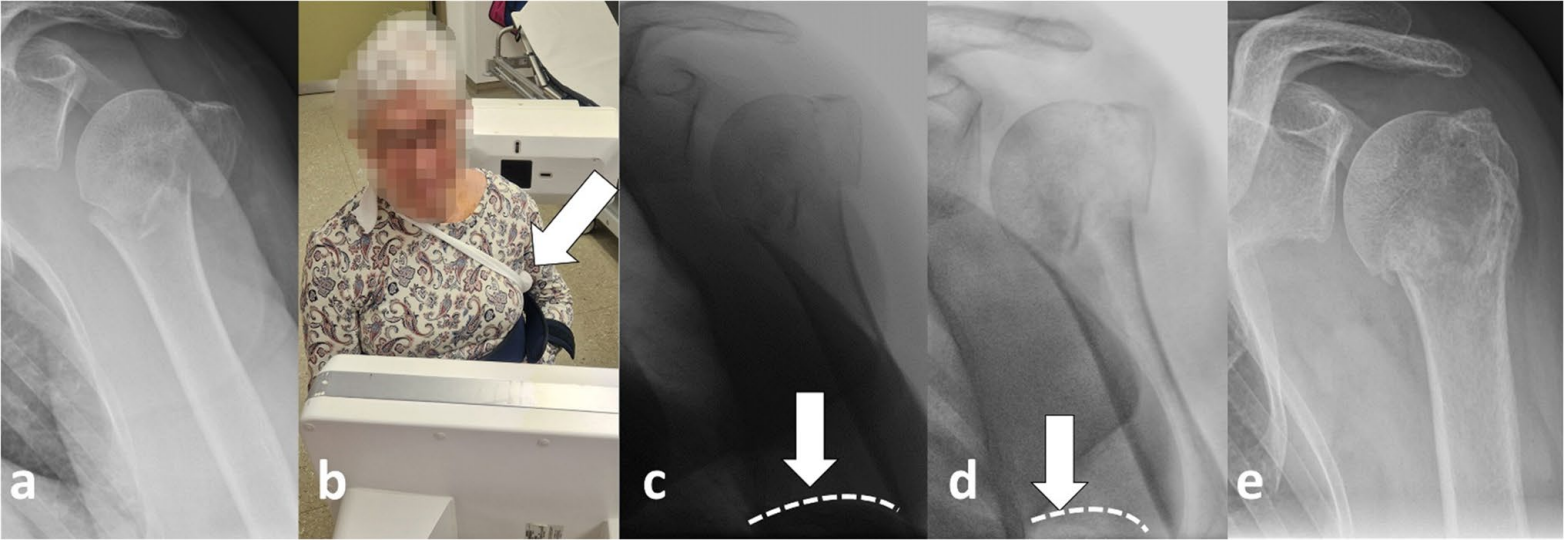
AP radiographs (a + e) and fluoroscopic images (c + d) of a 78-year-old patient with a valgus impacted PHF with medial shaft translation before (**a**), and immediately after (b + c) application of an arm sling with an axillary roll (white arrow and white dashed line). Fluoroscopic control after 3 weeks (**d**), and x-ray control after 9 weeks (**e**) showed a reduced medial hinge by about 5 mm and a reduced head-shaft-angle (HSA) by approximately 15° towads a nearly anatomic HSA

A so-called axillary roll can be placed under the axilla in order to serve as a hypomochlion to counteract medial displacement of the humeral shaft caused by traction of the pectoralis major muscle (Fig. 11). Such a roll can be made very simply using rolled towels or conventional elastic bandages, inserted into a tubular bandage and placed as far proximally as possible under the axilla. The free ends of the tubular bandage are lightly tightened and knotted at the contralateral neck region (Fig. 12) [4, 43].

It should be noted that the thicker the roll is wrapped, the greater the supposed hypomochlion effect may be, but the less proximal it can be placed in the axilla. The harder the roll is wrapped, the more sufficient the hypomochlion effect might be. However, this is at the expense of wearing comfort and patient compliance. Care should be taken to avoid pressure marks on the skin and tingling paraesthesia.This additional tool can be used in combination with all other aids mentioned here [4].

### Shoulder bag

An early change to a shoulder bag, ideally after 1–3 weeks, is reasonable for most of the PHFs. This tool is easy for patients to dress and undress. In addition, it offers comfort during the transition phase of regaining independence in daily life [4]. The shoulder bag is not mandatory and provides additional comfort for the patient. Care should be taken during application. The patient should be instructed to ensure that the shoulder bag is not applied too tight and short in craniocaudal direction in order to avoid axial impaction of the fracture, and to allow self-reducing through gravity in sitting and upright position.

### Hanging cast

**Fig. 13:**
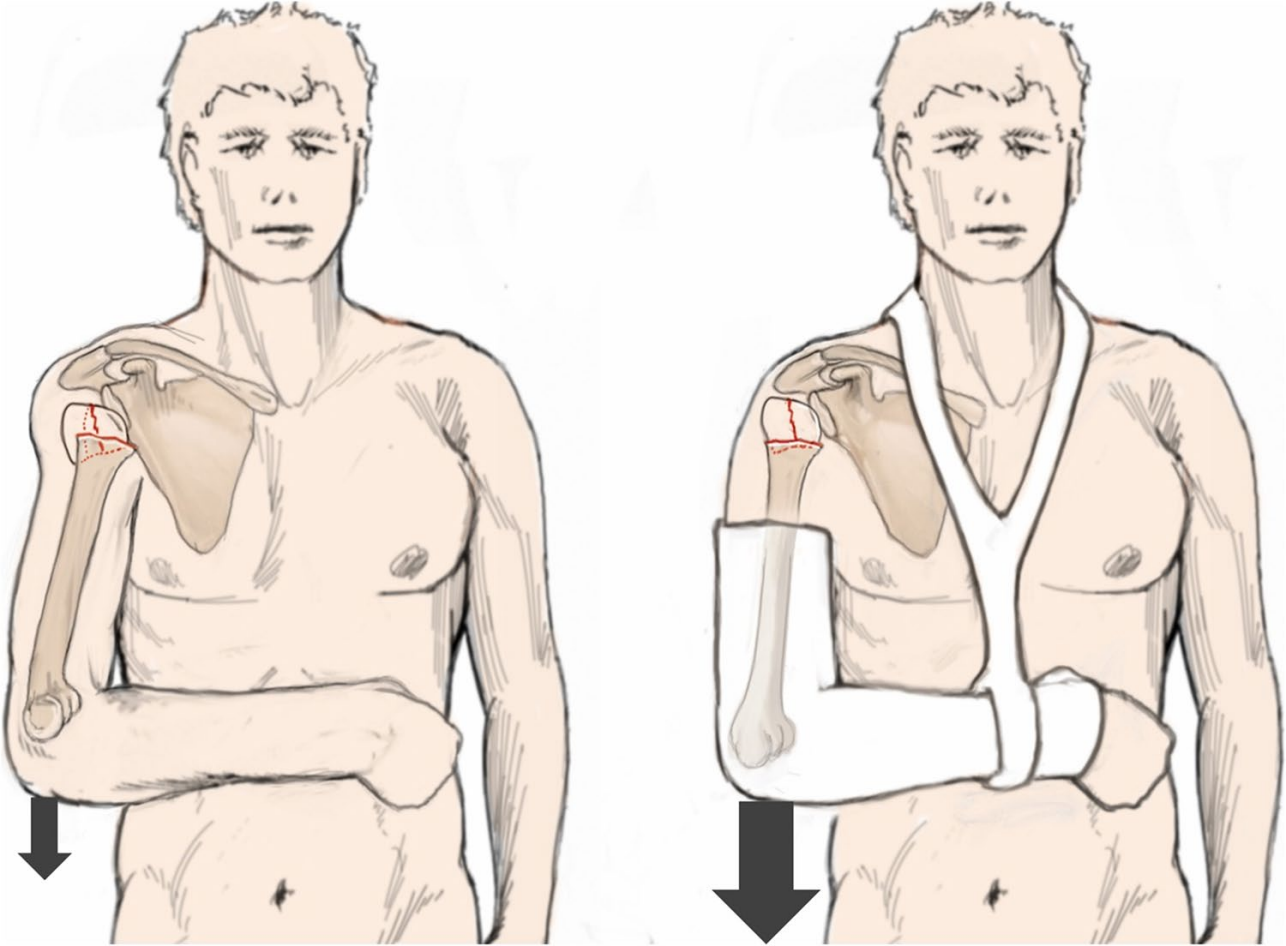
Patient with impacted fracture before (left) and after (right) application of a “hanging cast”. In this example, the weight of the cast acts as a counteracting force to the cranial displacing force of the deltoid muscle. Black arrow on the left figure shows limited traction force through the weight of the arm. Bigger black arrow on the right figure shows increased traction force through additional weight of the cast. Reprinted with changes from *“Algorithmus zur konservativen Behandlung von proximalen Humerusfrakturen”* (https://link.springer.com/article/10.1007/s11678-022-00702-y, Copyright© 2022, Razaeian) under the terms of the Creative Commons CC BY 4.0 license (http://creativecommons.org/licenses/by/4.0/deed.de)

**Fig. 14:**
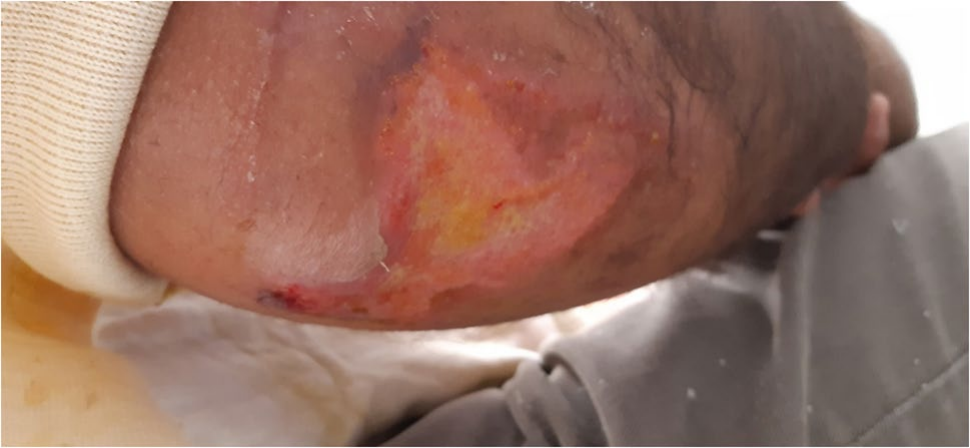
Cast-associated pressure point on the right olecranon as a possible complication of nonoperative treatment following application of a hanging cast. Sufficient padding of known vulnerable areas must be ensured. Reprinted from *“Algorithmus zur konservativen Behandlung von proximalen Humerusfrakturen”* (https://link.springer.com/article/10.1007/s11678-022-00702-y, Copyright© 2022, Razaeian) under the terms of the Creative Commons CC BY 4.0 license (http://creativecommons.org/licenses/by/4.0/deed.de)

A so-called “hanging cast” can be made by applying a circular plaster or U-shaped cast from the distal humerus over the 90° flexed elbow to the proximal humerus. Placing additional layers of plaster or even plastering small weight discs under the olecranon in the direction of the humeral shaft axis could help to reduce fracture shortening and impaction with the aid of gravity (Fig. 13). Caution is required in the elderly. In addition to skin pressure points on the elbow, choosing this tool can further complicate the social caring situation of this fragile patient group, which is already impaired by the fracture alone, and usually remain outpatient (Fig. 14). As a compromise, instead of heavy white plaster, much lighter and more comfortable synthetic cast can be used. However, this has the disadvantage of a lower traction effect [4].

### 4-phase aftercare scheme according to Krettek [43, 48]

An early functional 4-phase nonoperative aftercare regimen has been developed by Krettek that has proven itself for the majority of PHFs [43, 48]. The aim of this follow-up treatment is to restore shoulder function for daily activities with as little pain as possible, and thus quickly regain independence, which is essential in the elderly [4].

The four phases are defined as follows.

#### Phase 1 (week 1) Goal: immobilization, pain relief, and decongestion

##### Bandage/orthosis

Usually, Gilchrist bandage depending on the fracture type.

##### Exercises

Strict immobilization of the shoulder joint. The elbow, wrist and finger joints should be moved and exercised. The Gilchrist bandage can be opened to move the elbow joint. All activities should be carried out without pain or with only moderate pain.

##### Follow-up

One to two week after injury for clinical-radiological follow-up (X-ray: shoulder AP- and Y-view).

In the case of more complex fractures or if pain level does not decrease, an extension of phase 1 should be considered (no self-exercise, no physiotherapy).

##### Pain level on VAS-scale

Median of 5 points.

#### Phase 2 (week 2–3) Goal: pain consolidation and passive mobility

##### Bandage/orthosis

Ideally change to shoulder bag.

##### Exercises

Starting with pendulum exercises out of any bandage (Fig. 2), and guided passive movements using the contralateral arm.

The shoulder bag can be removed for body care. In order to carry out body hygiene, the axillary region can be accessed by tilting the trunk forwards or sideways. All activities should be carried out without pain or with only moderate pain. The exercises should not cause severe pain. The elbow, wrist and finger joints should still be moved and exercised.

##### Follow-up

3 weeks after injury for clinical-radiological follow-up (X-ray: shoulder AP- and Y-view).

Still only self-exercise, no physiotherapy.

##### Pain level on VAS-scale

Median of 4 points.

This phase usually represents a milestone for the patient, as the strict immobilization, which is perceived as restrictive, is lifted early, ideally after week 1 or 2. In addition, the patient can change to the much more comfortable shoulder bag and start the first pendulum exercises (Fig. 10).

#### Phase 3 (week 4–6) Goal: active mobility

##### Bandage/orthosis

Any aids/orthosis are partially or completly omitted.

##### Exercises

Continuing with pendulum exercises and guided movements. In addition, now the shoulder joint can also be actively moved (up to 90° of abduction and anteversion). All activities should continue to be carried out without pain or with only moderate pain. The elbow, wrist and finger joints should still be exercised.

##### Follow-up

6 weeks after injury for clinical-radiological follow-up (X-ray: shoulder AP- and Y-view).

Still only self-exercise, no assisted physiotherapy.

##### Pain level on VAS-scale

Median of 3 points.

#### Phase 4 (week 7–12) Goal: active mobility, full integration into daily life

##### Bandage/orthosis

Not applicable.

##### Exercises

In addition to passive and active movements, now also stretching exercises with a so-called shoulder rod and overhead exercises using a shoulder rope-pulley-set are recommended (available in medical supply stores). Physiotherapy can start now. Any stretching exercises are carried out up to the personal pain threshold (transition zone between comfortable and real pain). Start with external physiotherapy.

##### Follow-up

12 weeks after injury for clinical-radiological follow-up (X-ray: shoulder AP- and Y-view).

##### Pain level on VAS-scale

Median < 2 points.

Further radiographic follow-ups should be considered only in case of meaningful symptoms.

### Evidence on duration of immobilization (early versus delayed)

According to the latest Cochrane Review that also investigated the impact of early (usually one week after injury) versus delayed (three or more weeks) immobilization, available data was limited [12]. Only five trials with 350 participants made this comparison. Due to very low‐certainty evidence from these single trials, the authors were uncertain of the findings of better shoulder function at one year after early mobilization, and the findings of little or no between‐group difference in function at 3 or 24 months. In addition, there was very low‐certainty evidence of no relevant difference in quality of life at one year [12].

In a more recent randomized trial (RCT) that was published after the inclusion period of that Cochrane Review, 111 patients with a mean age of 70.4 years, and 66.7% of displaced fractures according to Neer were analyzed [49]. No significant differences were found in terms of function, and pain level at any time point independent of fracture pattern. In addition, no significant differences were observed in the complication rate, although there was a statistically nonsignificant trend towards a higher rate of secondary displacement after early mobilization (7.3% (4) vs. 1.8% (1)) [49]. The authors concluded to prefer only a short immobilization period of 1 week in order to not compromise patients’ independence, although also admitting that their study suffered relevant selection bias due to the lack of consensus on which fractures should be treated nonoperatively and which surgically, despite the setting of a single center study [49].

In the latest systematic review and meta-analysis that included also this study by Martinez, the authors analyzed six RCTs with a total of 470 patients [50]. There were no differences in patient-reported outcomes or pain at any follow-up except for a significant difference in combined function scores favoring the early mobilized group at 3 months follow-up [50]. In addition, there were no significant differences in the incidence of secondary fracture displacement and total complications [50].

Similar results were stated in the meta-analysis by Ataei. They observed superior function in early mobilized patients at 3 and 6, but not at 12 months of follow-up. Furthermore, there was no difference in pain level at 3, 6, or 12 months [51].

Considering complications, a recent review by Tunnicliffe did not reveal any relationship between complications and duration of immobilization, type of slings, or progressiveness of the follow-up treatment [52].

Taking into account this limited evidence, it should be mentioned that our proposed abovementioned practice is only a scheme. It may be useful to deviate from it in individual cases (e.g., isolated greater tuberosity fractures that occur less frequently in the elderly, severe shaft translation or varus/valgus displacement). In particular, the decisive transition from phase 1 to 2 and the start of initial exercises may need to be chosen later than shown here for more complex, unstable fractures. The practitioner may be faced with the dilemma of early release in order to avoid secondary stiffness or muscle atrophy on the one hand, and prolonged immobilization for pain relief and minimizing the theoretical risk of secondary displacement on the other hand, even if the latter concern might not be covered by the most recent randomized study [12, 49].

### Pain level monitoring

**Fig. 15:**
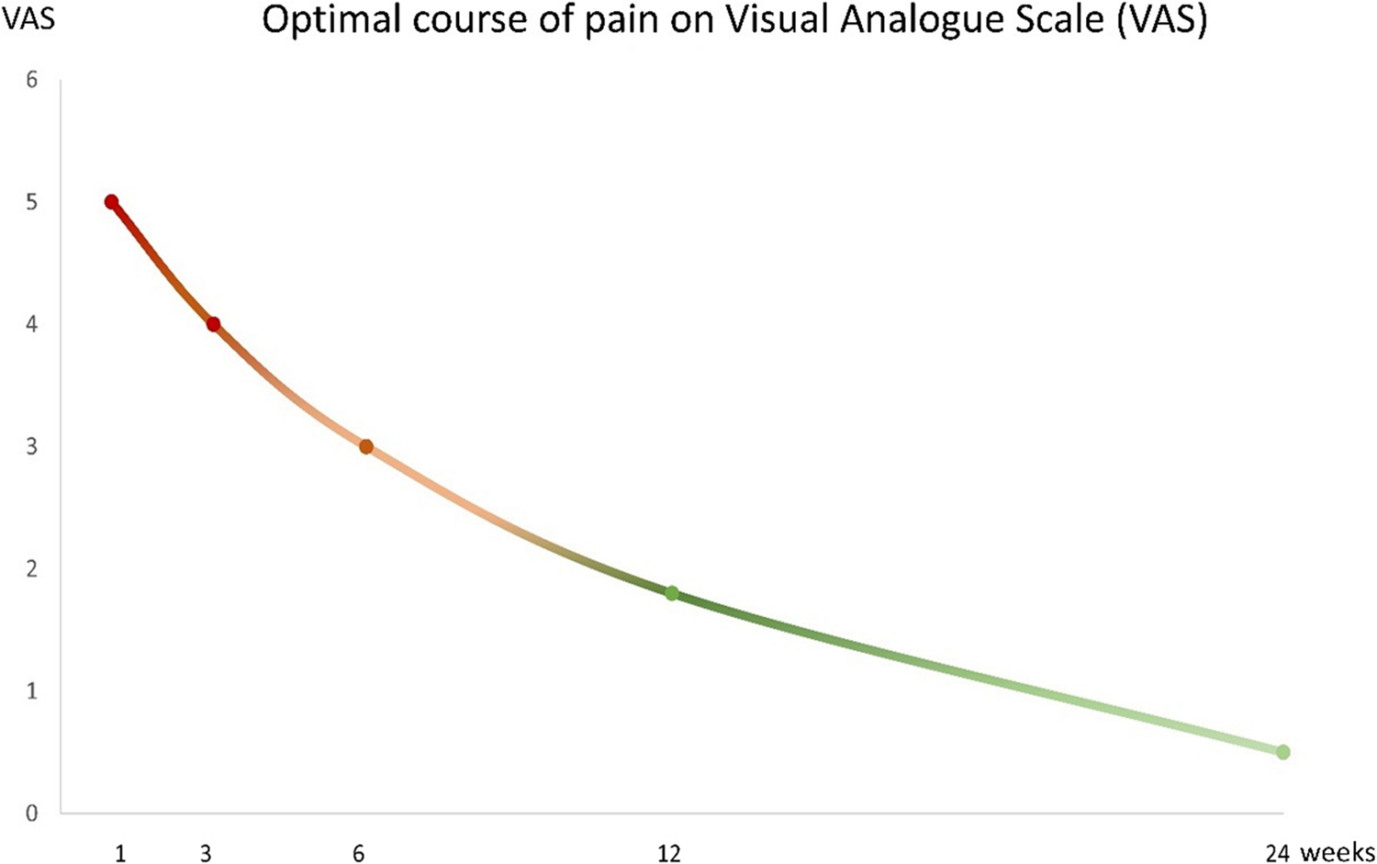
Schematic illustration of the optimal course of pain (VAS visual analog scale) in nonoperatively treated PHFs. Reprinted from *“Algorithmus zur konservativen Behandlung von proximalen Humerusfrakturen”* (https://link.springer.com/article/10.1007/s11678-022–00702-y, Copyright© 2022, Razaeian) under the terms of the Creative Commons CC BY 4.0 license (http://creativecommons.org/licenses/by/4.0/deed.de)

In our experience, pain level measured on the visual analog scale (VAS) is an important clinical parameter in the early phase of follow-up treatment [4]. Its intensity, and further development might predict the extent of fracture healing, and guide patients` pathway (Fig. 15) [4]. In the authors’ treatment algorithm, it also serves in decision-making process (Fig. 3) [4]. However, evidence for these assumptions do not yet exist, at least not for this type of injury [53].

In more complex fractures, additional investigation under fluoroscopic control with passive movement of the extremity might be helpful in order to assess fracture stability, and to release further exercises [4].

### Informed consent and patient-centered decision making

Informed consent is a crucial aspect that should be considered not only before any surgical but also nonsurgical treatment. Besides providing information about the current state of literature and possible complications, realistic treatment goals should be outlined in advance and, in the case of geriatric patients, relatives should be involved at an early stage in decision making process. These might be helpful in gaining a better overview of the patient's actual functional needs, comorbidities and social situation in case of mental impairment [4].

## Risks and management challenges

As a successful surgical treatment requires manual know-how, learning curve, and technical equipment, nonoperative treatment can also be far more complex and time consuming than simply applying a sling bandage [4]. As there is no strong consensus in treatment, close patient guidance is important in order to avoid unsettling patients, and relatives with divergent external second or more medical opinions during ongoing treatment. Ignorance, and strong conviction in one's own surgical skills [54] as well as economic pressure due to false incentives from reimbursement systems can lead to conflicting medical opinions. However, close patient guidance requires capacities for numerous outpatient follow-ups that not every clinic can provide. The same applies to qualified nursing staff with regard to the use of medical aids, and plaster bandages or technical equipment [4].

### Evidence on conversion to surgery and timing (acute versus delayed reverse shoulder arthroplasty)

*“These cases should teach the members of our profession to be kind, generous, and liberal towards each other; and not to impute to ignorance or inattention that which is the result of a generally incurable accident.”* Sir Astley Cooper (1839) [32, 55]

If pain level on the visual analogue pain scale does not decrease sufficiently and there is no timely increase in range of motion, conversion to operative treatment should be discussed. In a large prospective registry study with 31.761 primarily nonoperatively treated PHFs, early conversion rate to surgery within 60 days from fracture ranged only between 1.3% and 4.5% in the elderly [56]. In these scenarios of failed nonoperative treatment, reverse shoulder arthroplasty (RSA) has become an increasingly popular salvage procedure [57–60]. However, there is contradictory evidence on the impact of timing of RSA on patient outcomes, and complications [57–61]. Delayed RSA is defined in the majority of the literature as a period of 4 or more weeks after injury [57–61]. Table 2 shows an overview of studies comparing acute versus delayed RSA after failed initial nonoperative treatment.

**Table 2:**
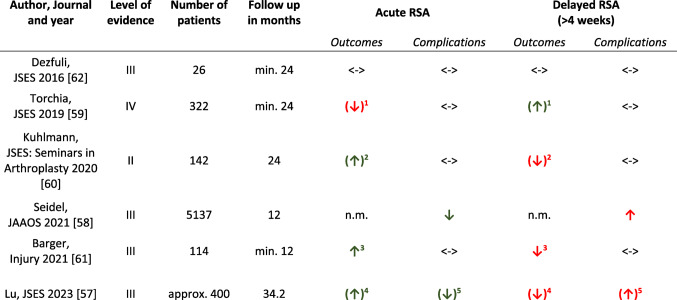
Overview of studies comparing acute versus delayed RSA after failed initial nonoperative treatment

*Min.* minimum, *Approx.* approximately, *N.m.* not measured. Horizontal arrows represent equality. Green, and red arrows represent superiority, and inferiority. Arrows in brackets represent limited results

^1^No statistically significant differences in forward flexion, and clinical outcome scores (ASES, CS, Simple shoulder test (SST), Penn shoulder, UCLA, SANE, and VAS score). Only 6° of more external rotation (*p* < 0.01) favoring delayed RSA

^2^Statistically significant difference in SPADI score (*p* < 0.05) favoring acute RSA. No further significant difference in range of motion (ROM), outcome scores (modified ASES, CS, SST-12, UCLA, and VAS score), and patient satisfaction

^3^Better DASH score (*p* = 0.034) and nonsignificant trend for better PROMIS physical function scores, and ROM favoring acute RSA

^4^Large heterogeneity of different outcome scores that were used. A subgroup analyses of 3 studies showed higher mean CS values (64.9 vs. 56.9 points) after acute RSA, although this difference did not reach minimal clinically important difference, and was not statistically significant after applying the Bonferroni correction

^5^Lower overall complication rate, and revision surgery favoring acute RSA, but with uncertainty about statistical significance as Bonferroni correction was not specified

In a recent systematic review and meta-analysis of retrospective observational studies on this topic, 6 studies with a total of over 400 patients were included comparing outcomes after failed initial nonoperative treatment [57]. These were subject to heterogeneity in the scope of different outcome scores that were used. However, a subgroup analyses of 3 of those studies showed higher Constant score mean values (64.9 vs. 56.9 points) after acute RSA, although this difference did not reach minimal clinically important difference, and was not statistically significant after applying the Bonferroni correction [57]. The same applied to range of motion, and complications. With regard to the latter, subgroup analyses revealed a lower overall complication rate (7.5% vs. 14.3%), need for reoperation (2.8% vs. 7.9%), and revision surgery (2.3% vs. 6.1%) in those treated with acute RSA [57].

 A large retrospective analysis by Seidel came to a similar conclusion [ 58 ]. Elderly patients aged 65 years and older with PHF who underwent primary RSA within a year of fracture were identified in a national insurance database from 2005 to 2014. Univariate one-year rates of revision and complication as well as surgery day cost of treatment were compared between acute (4,245 (82.6%)) and delayed RSA (892 (17.4%)) [ 58 ]. While acute RSA was associated with a higher surgery day cost (acute $15,770 ± $8,383, delayed $14,586 ± $7,271; *p*  < 0.001), delayed RSA resulted in a higher one-year revision rate (acute 1.7%, delayed 4.5%; *p*  < 0.001), and surgical complication rates of dislocation (acute 2.8%, delayed 6.1%; *p*  < 0.001) and mechanical complications (acute 1.9%, delayed 3.4%; *p*  = 0.007) [ 58 ].

Significant demographic and comorbidity differences existed between the both treatment cohorts. Acutely treated patients may have been older and had fewer comorbidities [58].

The authors admitted that patient characteristics might have affected the initial decision to attempt a trial of nonoperative treatment, and that although the authors aimed to control for this potential bias with multivariate analysis, there might be other unaccounted variables that caused differences they found [58].

Taking into account all this available evidence to date, we consider an observational “watch and wait” period of approximately 2–4 weeks as reasonable. This may vary depending on patient's will, fracture morphology, age, comorbidities, and pain level [4]. However, in our experience, fracture morphology, and purely radiological signs of secondary displacement should not function as sole criteria in decision-making process as it is known that residual bony deformities may be clinically well-compensated in the elderly [1, 4].

### Future outlook

Looking back at the available evidence of the last two decades and knowing that a majority of elderly patients with PHF might be successfully treated nonoperatively, it still remains unclear what exactly type of patient with what type of PHF would benefit from primary operative treatment.

**Fig. 16:**
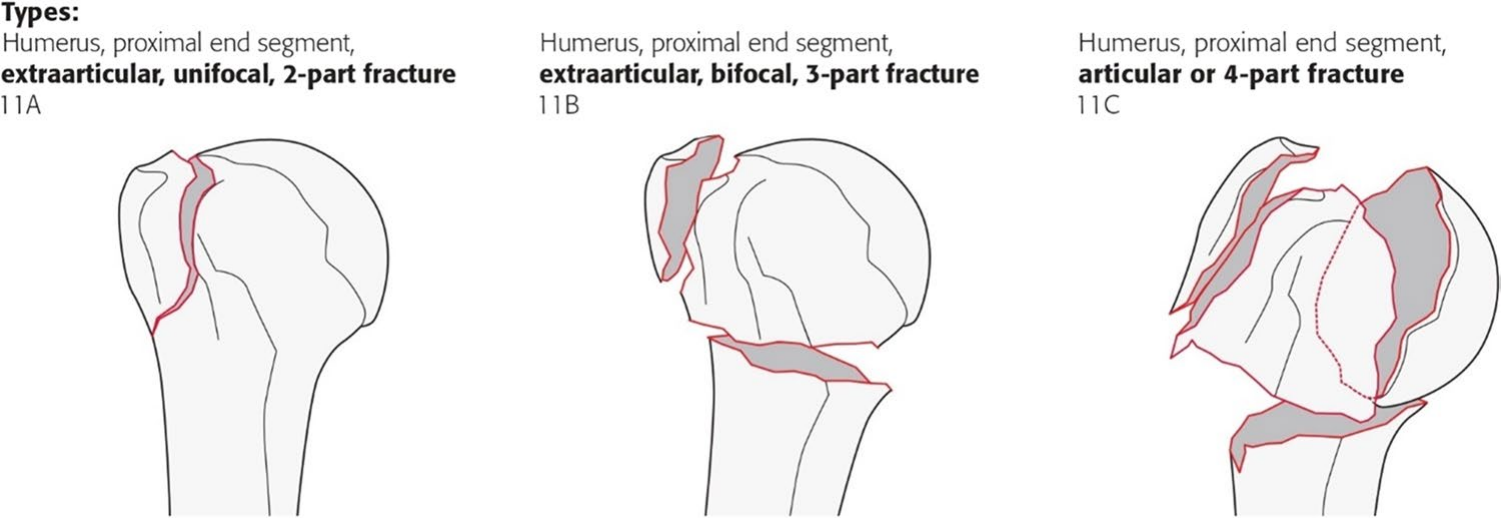
The illustration shows the three subgroups of the current AO/OTA 2018 classification system that attempted to incorporate the Neer classification into this system. The question arises how the exemplarily shown bifocal 11B1.1 fracture should be classified, when the surgical neck does not fulfill the displacement criteria according to Neer and only the greater tuberosity is displaced. In such a case, the fracture would not be a 3-part fracture, but a 2-part fracture according to the Neer classification system. However, this would automatically contradict the word “unifocal” and the illustrations shown in the type A group of the AO/OTA 2018 system [63]. Furthermore, it remains unclear how displacement is defined according to this classification system and its universal modifiers. Reprinted from Meinberg EG, Agel J, Roberts CS, et al. Fracture and dislocation classification compendium-2018. J Orthop Trauma 2018; 32 Suppl 1: S11-S20. ©2017 by AO Foundation, Davos, Switzerland; Orthopaedic Trauma Association, IL, US. Used with permission

In order to master the challenge of reaching a necessary consensus on this issue, the following unsolved problem areas might be useful to approach as prerequisites in future:

1. Lack of a reliable classification system as a common, unambiguous language for daily communication, and comparable characterization of patient cohorts in trials [5]. It is very unfortunate, for example, that attempts were made to incorporate the Neer classification into the new AO/OTA 2018 classification system for PHF [63]. Looking at the fracture descriptions (type A: extraarticular, unifocal, 2-part; type B: extraarticular, bifocal, 3-Part; type C: articular or 4-part) and the accompanying illustrations (Fig. 16) [63], it seems that Codman's rather than Neer's classification system is meant. This leads to critical inconsistencies in application of this new classification system, which were already adopted in recent comparative studies without pointing out these issues [64, 65]. For example, when in a bifocal 11B1.1 (new AO/OTA 2018, Fig. 16) that has three parts according to Codman only two of those three parts fulfill Neer`s displacement criteria (≥ 1 cm and/or 45°) this fracture has to be classified as a 2-part according to Neer`s classification system and not as a 3-part fracture as required in the new AO/OTA 2018 system [63]. Furthermore, such a fracture type would automatically contradict the word “unifocal” and the illustrations shown in the type A group (Fig. 16) [63], where it should have been allocated if the fracture descriptions of the AO/OTA 2018 system were consistently followed [63]. In addition, existence of so-called universal modifiers that indicate whether fracture displacement is present or not [63], without a more precise definition of displacement criteria, makes the whole issue more critical.
2. Lack of a reliable and valid outcome measurement tool specific for PHFs that allows reliable comparison of interventions across studies [66].
3. (If necessary, government-regulated) reduction instead of increase in treatment variability due to industry-triggered extension in different low-value surgical implants, and techniques (e.g., through national guidelines/directives and adjustment of adverse incentives in reimbursement system) [40]. In Denmark, for example, the introduction of an evidence-based national guideline has led to a decrease in surgery rate of PHFs [7].

These three problem areas might be expanded to include another controversial area of discussion in near future. Results of the eagerly awaited PROFHER-2 study could reveal the true significance of RSA in primary treatment of acute, displaced PHF in the elderly [67]. Soon, it will become clear whether there is a previously unproven outcome difference that significantly exceeds the minimal clinically important difference [14, 22, 68, 69], whether long-term complications typical for arthroplasty such as periprosthetic fracture, loosening and infection can be adequately unmasked within a usual 12 to 24 months follow-up in trials, or whether RSA is just another temporary parabolic trend that has replaced the hype about the locking plate in the elderly until the next innovation comes along [54, 70, 71].

Whatever the outcome, the significance of nonoperative treatment will remain relevant in future in view of upcoming high-quality trials [39, 67], national healthcare reforms including reimbursement systems that might change incentives for several indications as well as the international paradigm shift that we face, and that will force us to rethink away from volume-based towards value-based healthcare [54, 72, 73].

## Data Availability

No datasets were generated or analysed during the current study.
